# Severe Early-Onset Neonatal Listeriosis: An Unusual Diagnosis

**DOI:** 10.7759/cureus.92005

**Published:** 2025-09-10

**Authors:** Marta Rodrigues Amaral, Francisca Strecht Guimarães, Margarida Ribeiro, Catarina Maia, Fátima Menezes, Liliana Quaresma

**Affiliations:** 1 Pediatrics and Neonatology, Unidade Local de Saúde Entre Douro E Vouga, Santa Maria da Feira, PRT; 2 Gynecology and Obstetrics, Unidade Local de Saúde Entre Douro E Vouga, Santa Maria da Feira, PRT

**Keywords:** brain injury, congenital infection, early-onset sepsis, listeria monocytogenes, neonatal listeriosis

## Abstract

*Listeria monocytogenes* is a Gram-positive bacterium with a low incidence of diagnosed infections. Maternal-fetal transmission can generate severe conditions in neonates manifested as sepsis, often clinically indistinguishable from other causes of neonatal sepsis, and is a particularly important cause of meningitis. Herein, we report a case of a 31-week and 3-day preterm male infant, weighing 1545 g (P10-50, Fenton chart), with Apgar scores of 0, 4, and 6 at 1, 5, and 10 minutes, respectively, who required advanced resuscitation and ventilatory support. He presented with severe respiratory distress and hemodynamic instability. Diagnosis was made by isolation of *Listeria monocytogenes* from the newborn’s blood culture and molecular testing of the placenta using polymerase chain reaction (PCR). Prompt empiric antibiotic therapy with ampicillin, gentamicin, and cefotaxime was initiated and later adjusted based on microbiological findings, leading to complete recovery of the neonate. This case illustrates the diagnostic challenges of early-onset listeriosis, which often presents with non-specific signs in clinically unstable preterm infants. This presentation reinforces the need for a high index of clinical suspicion and appropriate microbiological and molecular investigations.

## Introduction

*Listeria monocytogenes* (LM) is a Gram-positive bacterium responsible for listeriosis, a potentially serious infection usually transmitted through contaminated food. Although rare, listeriosis can cause severe illness, especially among vulnerable groups such as newborns, pregnant women, elderly individuals, and immunosuppressed patients [1]. Pregnant women are particularly at higher risk for listeriosis, but symptoms are non-specific and diagnosis is difficult. Although the maternal illness is usually mild, neonatal illness is frequently severe and potentially fatal, with mortality rates ranging from 20% to 60% among diagnosed patients [2]. For neonatal infection acquired from maternal transmission, maternal infection typically occurs 2 to 14 days before delivery. However, the incubation period for invasive listeriosis in adults, including pregnant women, can be relatively long, typically ranging from 3 to 70 days after exposure. LM has the ability to infect the placenta and cross the placental barrier, potentially causing miscarriage, stillbirth, premature birth, or neonatal listeriosis [3,4].

Neonatal listeriosis presents in two distinct forms: an early-onset form, due to transplacental transmission, and a late-onset form typically associated with exposure to the pathogen at the birth canal or, less likely, a nosocomial transmission [5,6]. Early-onset listeriosis develops within the first six days of life, often leading to sepsis and pneumonia. This form is especially common among preterm infants and is usually associated with chorioamnionitis. Late-onset listeriosis typically occurs between 7 and 28 days after birth, and commonly manifests as meningitis. Early-onset listeriosis is associated with a higher risk of severe complications and mortality [7,8].

This case provides a relevant clinical example of severe early-onset neonatal listeriosis and highlights the need to maintain a high index of suspicion even in the absence of clear maternal risk factors. It further illustrates the value of multidisciplinary coordination, particularly between obstetricians, neonatologists and microbiologists to achieve timely diagnosis and improve neonatal outcomes.

## Case presentation

We report a case of a preterm neonate born via emergency cesarean section at 31 weeks and 3 days of gestational age due to spontaneous labour with fetal distress. The 35-year-old mother, G2P1A1 (second pregnancy, with one prior live birth and one miscarriage), had a history of a first-trimester spontaneous abortion in a previous pregnancy. The current pregnancy had been regularly monitored and was uneventful until the day prior to delivery, when she presented with uterine contractions, decreased fetal movements, and fever. She was transferred in utero from a level II hospital to our center in anticipation of preterm labor. Tocolysis and an incomplete course of antenatal corticosteroids were initiated. Group B Streptococcus colonization status was unknown.

Intraoperatively, rupture of membranes occurred with meconium-stained amniotic fluid. A male newborn weighing 1545g (P10-50, Fenton chart) was delivered in apparent stillbirth and received advanced neonatal resuscitation, with endotracheal intubation, chest compressions, and administration of endotracheal epinephrine. Apgar scores were 0, 4, and 6 at 1, 5, and 10 minutes, respectively.

**Figure 1:**
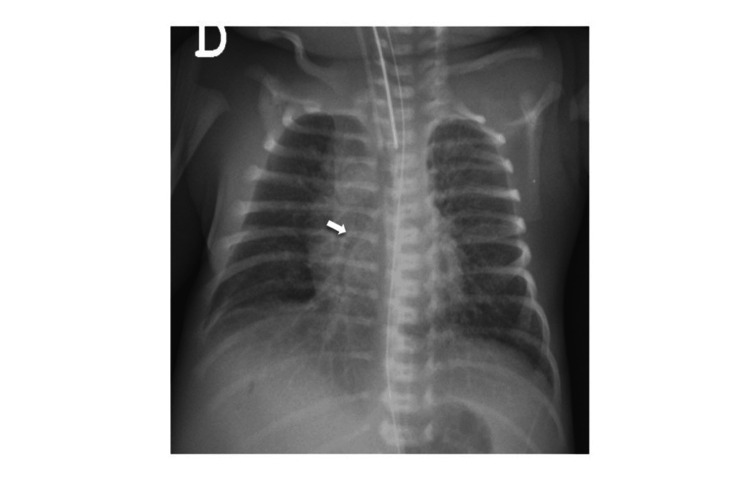
Chest radiograph, anteroposterior incidence, showing a diffuse granular pattern, with air bronchogram (white arrow), and no signs of pleural effusion or pneumothorax.

The infant was admitted to the Neonatal Intensive Care Unit. On admission, he was pale, hypotonic, and hypoactive. Initial chest radiograph showed a diffuse granular pattern consistent with congenital pneumonia (Figure 1). He required invasive mechanical ventilation and received surfactant replacement therapy. The patient remained hemodynamically unstable and required inotropic support with dopamine for three days, at a maximum dose of 7 mcg/kg/min.

Laboratory investigations revealed leukopenia, elevated C-reactive protein and markedly elevated procalcitonin. Blood gas analysis indicated metabolic acidosis requiring administration of two boluses of normal saline (Table 1).

**Table 1:** Blood test results

Parameter	Result	Reference Range
Hemoglobin	16.8 g/dL	14.5 – 22.5 g/dL
Leukocytes	4,300 /μL	9,000 – 36,000 /μL
Platelets	180,000 /μL	150,000 – 450,000 /μL
C-reactive protein	82.3 mg/L	< 5 mg/L
Procalcitonin	45.2 ng/mL	< 0.5 ng/mL
pH	7.17	7.32 – 7.42
HCO₃⁻	17.5 mmol/L	22 – 26 mmol/L
pCO₂	48 mmHg	41 – 51 mmHg
Base excess	-11	±2 mmol/L
Lactate	10.7 mmol/L	< 2 mmol/L

Blood culture was collected, and empiric triple antibiotic therapy with ampicillin (100 mg/kg/dose every 8 hours), gentamicin (4.5 mg/kg/dose every 36 hours), and cefotaxime (50 mg/kg/dose every 8 hours) was initiated. Due to clinical instability, lumbar puncture could not be performed.

The newborn was extubated after 36 hours, then was placed on nasal continuous positive pressure for 36 hours, showing favorable clinical progression. Blood culture became positive after 15 hours of incubation, with subsequent isolation of *Listeria monocytogenes*, sensitive to penicillin and ampicillin. In the further course, cefotaxime was discontinued after eight days of treatment, and antibiotic treatment was continued with ampicillin and gentamicin for 14 days, followed by ampicillin monotherapy to complete a 21-day course. C-reactive protein (CRP) levels were monitored and decreased to normal value by day 9 of life.

Furthermore, placental pathology examination revealed acute placentitis associated with acute chorioamnionitis, along with acute villitis and parenchymal abscess formation. Molecular testing using PCR confirmed the presence of *Listeria monocytogenes*.

The mother denied ingestion of unpasteurized dairy products, undercooked meat or fish, or unwashed produce. Serologic testing for *Listeria monocytogenes* (performed on maternal serum obtained at the time of delivery) was positive for serogroups 1/2a and 4b. Maternal blood culture collected 21 days after delivery was negative.

**Figure 2:**
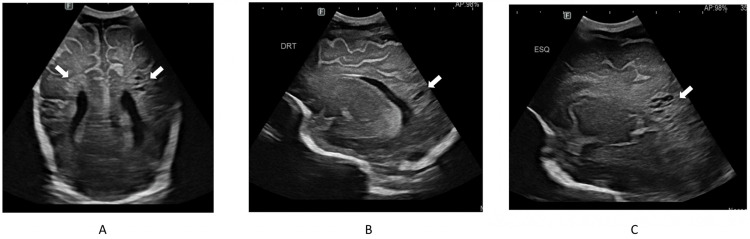
Coronal (A) and parasagittal (B,C) cranial ultrasound scans showing bilateral parieto-temporo-occipital porencephalic cysts (white arrows).

Throughout the clinical course, the newborn remained in good condition. Serial cranial ultrasounds revealed persistent hyperechogenicity in the frontal, periventricular, and occipital lobes, which resulted in the formation of bilateral parieto-temporo-occipital porencephalic cysts. These findings are illustrated in Figure 2A-2C. Cranial magnetic resonance imaging at term-equivalent age confirmed multicystic bilateral parieto-temporo-occipital leukoencephalomalacia, grade III periventricular leukomalacia, ventricular dilatation, and diffuse thinning of the brainstem.

On the 43rd day of life, the patient was discharged healthy and remained under follow-up in neonatology consultation, physiotherapy and occupational therapy sessions. At seven months of age, cranial ultrasound showed indirect signs suggestive of cerebral atrophy. Clinically, the infant demonstrated adequate weight gain and normal psychomotor development. Neurological assessment revealed mild axial and limb hypertonia, more evident in the upper limbs, but otherwise normal findings.

## Discussion

This case highlights a typical presentation of early-onset neonatal listeriosis, associated with prematurity, sepsis, and pneumonia, often clinically indistinguishable from other causes of neonatal sepsis. Wu et al. reported that the most common antepartum findings were maternal fever (93%), meconium-stained amniotic fluid (86%), and intrauterine fetal distress (79%) [9]. As similarly described in multiple studies, the presence of prematurity and meconium-stained fluid should always raise clinical suspicion for neonatal listeriosis [10-11]. The combination of meconium-stained amniotic fluid, fetal distress, and early-onset symptoms supports a transplacental infection consistent with previous reports [12-13].

Maternal serologic testing was positive for *Listeria monocytogenes* serogroups 1/2a and 4b. Among the 13 known serotypes, only four (1/2a, 1/2b, 1/2c, and 4b) are commonly linked to human infection. Serotype 4b is especially associated with pregnancy-related cases, likely due to its increased ability to cross the placenta [7].

Our preterm neonate presented with severe systemic compromise at birth, including hypotonia, apnea, cyanosis, bradycardia, and the need for advanced resuscitation. These findings are consistent with those reported by Wu et al.’s observations, in which most infected neonates were premature and showed respiratory distress requiring intubation [9]. Similarly, in the MONALISA cohort, 57% of infants were born prematurely and 22% before 32 weeks, consistent with our patient’s gestational age. At birth, 70% of neonates in the cohort exhibited abnormal clinical status, including 56% with respiratory distress, 35% requiring mechanical ventilation, and 8% needing inotropic support [12].

Laboratory parameters in our case indicated severe infection, with leukopenia, a CRP of 82.3 mg/L, and procalcitonin of 45.2 ng/mL. Similar results were described by Wu et al., who reported elevated CRP in 93% and leukocytosis or leukopenia in most cases [10]. Raimundo et al. similarly documented a rapid inflammatory response requiring early hemodynamic intervention [10].

Histopathological analysis of the placenta in our case confirmed acute placentitis with abscess formation, which is a hallmark of congenital listeriosis, as also emphasized by Teixeira et al., who highlighted placental examination as essential for diagnosis [14]. Placental cultures are considered the gold standard for confirming maternal-fetal listeriosis, with greater sensitivity than maternal blood cultures (80% vs. 55%). Placental biopsies permit histopathological examination, which can reveal hallmark signs of Listeria infection, such as microabscesses, chorioamnionitis, and inflammatory infiltrates, even in the absence of positive cultures [7].

Neuroimaging revealed bilateral multicystic leukoencephalomalacia, ventricular dilatation, and brainstem thinning. Similar findings, such as ventriculomegaly, periventricular hemorrhages, and leukoencephalomalacia, have been reported, frequently correlating with poor neurological outcomes [9,13]. In our case, early neuroimaging revealed severe findings, including multicystic leukoencephalomalacia. By seven months (five months of corrected age), despite the infant had achieved some developmental milestones and maintained adequate weight gain, he showed mild axial and limb hypertonia.

As demonstrated by Anand et al., Listeria may induce brain injury via hematogenous spread and inflammation, even in the absence of direct CSF invasion [15]. In our case, lumbar puncture was not performed due to the patient’s initial clinical instability, making it impossible to confirm or exclude meningitis. Given the severity of the presentation, a decision was made to extend ampicillin therapy to 21 days. The observed multicystic leukoencephalomalacia could be attributed to direct infection-related brain injury or to perinatal asphyxia, associated with the underlying intrauterine infection.

Treatment of listeriosis is based on high-dose ampicillin, which is effective as monotherapy. However, gentamicin is often added for its potential synergistic effect, despite limited evidence. This combination is frequently used in clinical practice to increase antimicrobial efficacy, particularly in severe cases with systemic involvement [9,16]. The duration of therapy varies depending on the severity of the infection and the patient’s overall health. Cephalosporins are ineffective against *Listeria monocytogenes* due to intrinsic resistance. Charlier et al. found that maternal antibiotic treatment for at least one day before delivery significantly reduced neonatal severity, although this was not applicable in our case, early postnatal treatment likely contributed to the favorable outcome [12].

Although the infant showed some early developmental gains, neurological follow-up revealed evolving motor abnormalities. Nevertheless, the long-term prognosis in neonatal listeriosis remains uncertain and highly variable as highlighted in reports [9,12]. Ongoing neurodevelopmental surveillance and access to early intervention services are critical.

## Conclusions

Maternal-neonatal listeriosis is a rare infection with severe presentation in neonates, as illustrated by the case we describe, which required intubation and ventilatory support. This case highlights the importance of early recognition of neonatal listeriosis, particularly in preterm infants presenting with respiratory depression, meconium-stained amniotic fluid, and a history of maternal fever.

Prompt treatment of this condition is crucial to improve neonatal outcomes. As a pregnancy-related disease, prevention is the best strategy to reduce morbidity. Dietary guidance for pregnant women is necessary to strengthen food safety awareness and help them avoid consuming contaminated food. So it is essential that obstetricians and neonatologists be aware of this infection and consider it in their differential diagnosis.
